# Interlinkages between Climate Change and Food Systems: The Impact on Child Malnutrition—Narrative Review

**DOI:** 10.3390/nu15020416

**Published:** 2023-01-13

**Authors:** Carlo Agostoni, Mattia Baglioni, Adriano La Vecchia, Giulia Molari, Cristiana Berti

**Affiliations:** 1 Pediatric Area, Fondazione IRCCS Ca’ Granda Ospedale Maggiore Policlinico, 20122 Milan, Italy; 2 Department of Clinical Sciences and Community Health, University of Milan, 20122 Milan, Italy; 3 Action Contre la Faim (ACF-France), CEDEX, 93558 Montreuil, France

**Keywords:** children, undernutrition, obesity, climate change, COVID-19, food system, breastfeeding, complementary feeding, sustainable nutrition, socioeconomic inequalities

## Abstract

The pandemics of obesity, undernutrition, and climate change represent severe threats to child health. They co-occur; interact with each other to produce sequelae at biological, psychological, or social levels; and share common underlying drivers. In this paper, we review the key issues concerning child diet and nutritional status, focusing on the interactions with climate and food systems. Inadequate infant and young child feeding practices, food insecurity, poverty, and limited access to health services are the leading causes of malnutrition across generations. Food system industrialization and globalization lead to a double burden of malnutrition, whereby undernutrition (i.e., stunting, wasting, and deficiencies in micronutrients) coexists with overweight and obesity, as well as to harmful effects on climate. Climate change and the COVID-19 pandemic are worsening child malnutrition, impacting the main underlying causes (i.e., household food security, dietary diversity, nutrient quality, and access to maternal and child health), as well as the social, economic, and political factors determining food security and nutrition (livelihoods, income, infrastructure resources, and political context). Existing interventions have the potential to be further scaled-up to concurrently address undernutrition, overnutrition, and climate change by cross-cutting education, agriculture, food systems, and social safety nets. Several stakeholders must work co-operatively to improve global sustainable nutrition.

## 1. Introduction

Improvements in survival, nutrition, and education have been achieved in recent decades. Nonetheless, the world is far from achieving the Sustainable Development Goals to eliminate malnutrition and poverty by 2030 [1]. Child malnutrition is still of public health concern. Only one-quarter of countries are on track to meet the targets on stunting, wasting, and overweight [1]. Furthermore, pediatric populations are threatened by a double burden of malnutrition whereby undernutrition coexists with overweight, obesity, and other diet-related non-communicable diseases (NCDs). Along with the burden of malnutrition, today’s children are facing uncertainty for the future, with environmental change, conflicts, the COVID-19 pandemic, and inequalities threatening their health and well-being [2,3]. In 2015, countries committed to the Paris Agreement in an attempt to limit warming to below 2 °C; nevertheless, global carbon dioxide (CO_2_) emissions continue to rise steadily. Climate change negatively impacts health determinants (e.g., agriculture and access to water) at the global level. These effects affect populations who might be barely accountable for the problem, thus exacerbating issues between countries associated with social and economic inequality [4]. The global food system has failed to substantially improve the second sustainability goal on hunger, mainly due to poor management and distribution, with the frailty of the system highlighted by the COVID-19 pandemic. Moreover, the food system is one of the most important contributors to greenhouse gas (GHG) emissions, accounting for 20–30% of global GHG emissions [5].

Herein, we review the key issues relating to child malnutrition with a particular focus on climate change and food systems.

## 2. The Double Burden of Child Malnutrition: Epidemiology and Feeding Practices

Malnutrition in all its forms occurs in an intergeneration cycle [6] and is the leading cause of poor health globally [7]. Early-life undernutrition exerts short-term effects on both morbidity and mortality and lifelong effects on overweight, obesity, and/or NCDs [6,8]. Despite a decline during the 2000–2020 period, 149 million children under the age of 5 years suffer from stunting as a result of chronic or recurrent undernutrition [1], with undernutrition from conception to the 2nd year of life representing one of the major contributors [9,10,11,12]. Stunting can exert long-term effects on individuals and societies, including poor cognition and educational performance, low adult wages, and lost productivity [13,14,15]. In 2020, 45.4 million children under 5 years suffered from wasting because of acute food shortages and/or infections. This condition is associated with weakened immunity, increased risk of infectious diseases, and death. At least 340 million children under 5 years suffer from micronutrient deficiencies (i.e., hidden hunger), with the highest prevalence in LMICs [6], and adverse impacts on growth and development, immunity and tissue development, and risk of death [16]. Hidden hunger may also occur in the absence of an energy-deficient diet; thus, an obese child can suffer from micronutrient deficiencies, as modern diets are energy-dense but nutrient-poor. Child overweight and obesity imply multiple medical, psychological, and social comorbidities, which lead to reduced quality of life, increased social disadvantages, and the transmission of risks across generations [17,18]. Whereas rates have plateaued at high levels in high-income countries (HICs), the proportion of overweight in children under 5 years has risen in low- and middle-income countries (LMICs) from 33 million in 2000 to 39 million in 2020 as a result of the increased availability of “cheap calories” from ultra-processed foods [16].

**Figure 1:**
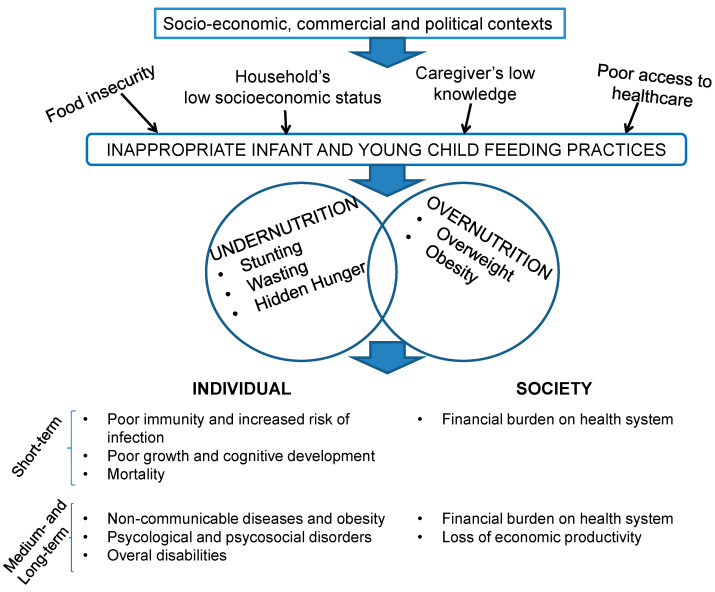
Poor infant and young child feeding practices: drivers and impacts on individual and societal health in the short and long term.

Amongst the common modifiable and interrelated drivers shared by the different forms of malnutrition, poor infant and young child feeding (IYCF) practices likely play a crucial role, as the first months of life represent the first opportunity to influence the offspring’s health and potential [19]. Figure 1 summarizes the main determinants and the overall lifelong consequences of inappropriate child feeding practices.

Whereas international authorities have set recommendations for IYCF [20], breastfeeding rates and duration remain suboptimal across the globe (only 42% of children under the age of 6 months are breastfed) [6,16]. Conversely, total worldwide commercial milk-based formula sales increased by 115% between 2005 and 2019, from 3.5 to 7.4 kg/child [21], and complementary feeding is introduced to infants aged 4 months or younger [22,23].

Each year, approximately 975,000 cases of childhood obesity can be attributed to not breastfeeding according to recommendations [24]. Inappropriate marketing of food products that compete with breastfeeding adversely affects a mother’s decision to breastfeed. The International Code of Marketing of breast-milk Substitutes was first adopted in 1981 and subsequently updated many times; however, the implementation of the code is not sufficient for the improvement of IYCF [25].

Complementary foods are often nutritionally inadequate (worldwide, 29% of children aged 6–23 months eat foods from the minimum number of food groups) [6,16], with potential implications for stunting and overweight in low-income countries (LICs) and HICs, respectively. In recent decades, changes brought about by the industrialization and globalization of food systems [26] have led to marked and rapid shifts in contemporary food systems towards a global diet dominated by a higher intake of ultra-processed foods (nutritional transition). This trend has likely contributed toa widespread obesogenic environment [27,28]. Concurrently, ultra-processed products might contribute to stunting and micronutrient deficiencies by replacing more nutritious whole foods [7,28].

Poor diets and resulting malnutrition are among the major present societal challenges. They are not solely a matter of personal choices but depend on several socioeconomic, commercial, and political factors and shocks [29]. The affordability of foods is a key determinant of dietary patterns and related nutritional outcomes [30]. Most people cannot access or afford a healthy diet or quality nutrition care. Nutrient-dense foods such as fresh fruits and vegetables may have a high “per calorie” cost compared to calorie-dense foods low in nutritional value, whereas calorie-dense processed foods are characterized by a relatively low cost [31], suggesting an impact of socioeconomic inequalities on the rates of malnutrition. In LICs, most animal-sourced foods and fortified infant cereals are expensive [30]. Relative prices of dairy products and eggs are strongly associated with stunting rates. For example, a 1-SD increase in milk prices was associated with a 2.8 percentage-point increase in the prevalence of stunting [30]. Stunting prevalence tends to be more pronounced in association with the lowest socioeconomic status or household wealth status [9,32,33]. Likewise, women and children in lower socioeconomic groups seem to show high levels of obesity compared with the rest of the population [34,35]. Overall, poverty likely predisposes low-income individuals towards a suboptimal diet. Indeed, minimum diet diversity is considerably lower for children in the poorest households, rural areas, or with a less-educated mother [36].

## 3. Climate Change Interactions with the Agro-Ecosystem and Effects on Child Health

The effects of climate change are expected to amplify health challenges for human beings, the environment, and the planet. Climate change may influence food production and nutrition security due to its impact on the agro-ecosystem [37]. Effects on quantity, quality, access to, and affordability of foods from agricultural, fishery, and livestock sectors exacerbate nutrient deficiencies, chronic undernutrition, and vulnerability among the most food-insecure population groups [4,7,38]. Furthermore, current changes in climate are influenced by food systems and modern consumption patterns.

With respect to obesity and undernutrition, climate change constitutes a global syndemic, i.e., a synergy of epidemics that co-occur; interact with each other to produce complex sequelae at biological, psychological, or social levels; and share common underlying societal drivers [7]. Undernutrition is the largest threat to health as a result of climate change. Climate change may worsen the nutrient value of foods. Trends in climate suitability for disease transmission are also particularly concerning, with children among the most susceptible [4,39]. A link between increasing temperature and obesity has also been also suggested due to both a reduction in physical activity and the effects on fruit and vegetable production, leading to increased prices and shifts towards cheaper processed food and beverages [7].

### 3.1. Effects on the Agro-Ecosystem, Food and Nutrition Security, and Livelihoods

Global warming may destabilize the ecosystem by leading to lower crop yields, livestock production, and fish catch, as well as a decrease in forest ecosystem services.

Its effect has already negatively affected the worldwide yields of basic crops that are essential for food security such as wheat and maize [40] and will continue to do so, even under low levels of warming [40]. Projections estimate that by 2100, the impact of climate change on crop yields for high-emission climate scenarios will range between −20 and −45% for maize, between −5 and −50% for wheat, between −20 and −30% for rice, and between −30 and −60% for soybean [41]. Elevated levels of CO_2_ have been shown to decrease protein concentrations in wheat, barley, rice, and potato crops. Furthermore, climate change may favor the spread of pests that threaten crop production, allowing them to appear earlier and in areas where they could previously not establish [42]. An example is the desert locust outbreak across several East African countries between 2019 and 2020, which originated in the Arabian Peninsula in the aftermath of the powerful tropical c Mekunu [43]. Climate change is also predicted to increase foodborne and waterborne diseases, changing microbial communities and species interactions, [44,45,46]. The WHO estimates that climate change will cause an additional 48,000 deaths in children aged under 15 years due to diarrheal disease by 2030 and an additional 330,000 deaths by 2050 [47].

Dramatic effects also occur concerning livestock productivity, animal health, and biodiversity. Recent studies have shown that dairy cows under heat stress in southern European countries are associated with an estimated milk loss of up to 5.5 kg/cow/day and that in sub-Saharan countries, 20–60% losses in animal numbers were recorded during the past two decades due to serious drought [41]. Likewise, warming affects fisheries by modifying the chemical composition of the aquatic environment such as salinity content, oxygen concentration, and acidification. These changes may decrease the maximum body weight of fish species and result in lower catch potentials [48,49]. Moreover, warming likely leads to reduced long-chain polyunsaturated fatty acid content iron contents in seafood.

Another significantly alarming factor is the threat posed to forests and trees by climate change, considering their paramount roles in the ecosystem, i.e., delivery of a clean and reliable water supply, protection against landslides and land degradation, provision or enhancement of the habitat of terrestrial and aquatic animals, and the provision of a range of products for household use or sale. Studies have shown that warming and changes in precipitation have resulted in a spike in tree mortality [50] and that tree species loss will lead to reduced potential in terms of functions within the ecosystem [51,52].

### 3.2. Increasing Recurrence of Extreme Weather Events

Because of global warming, extreme weather events, including wildfires, floods, storms, and droughts, are becoming increasingly frequent worldwide. During the period of 2016–2019, there an increase in the risk of wildfires was observed in several countries, with the largest impact on Lebanon, Kenya, South Africa, Australia, and the USA. In mainland Southeast Asia, the occurrence of floods and the maximum magnitude of floods significantly increased from 1985 to 2018 [53]. In 2018, Europe, the eastern Mediterranean region, and, specifically, Mongolia experienced unusually long periods of consecutive months under excess drought [5]. Recurrent and more prolonged droughts affect LMICs, i.e., East Africa, Asia, and the Pacific area. In Asia, by 2100 compared to 1990, rice yields could be reduced by as much as 50%, and wheat and maize crop yields could be reduced by 30%. Furthermore, an additional 38 million people in Asia and the Pacific are likely to be pushed to hunger by 2030 [54]. Likewise, up to 60% of cereal production in Somalia is below average, and almost 10 million livestock have died [54].During the last two decades, Africa has also experienced tropical storms [55]. In 2019, cyclones Idai and Kenneth displaced millions of people across Mozambique, Malawi, and Zimbabwe and destroyed thousands of hectares of crops, seed stock, fisheries, and infrastructure, severely impacting access to food [54]. Tropical storms, hurricanes, and typhoons also seriously affect Latin America and the Caribbean [56]. Since 2012, the region has been affected more frequently by droughts and cyclones, causing more than 60 million more people to be food insecure in 2020 than in 2019 [54]. These weather conditions have caused a coffee crisis in Honduras and Guatemala; the loss of close to 80% of the maize harvest in Guatemala; and a 50% drop in harvests of sorghum, sunflower, and corn in Mexico. In Haiti, similar cycles of weather conditions in 2020 and 2021 significantly reduced food production, becoming one of the key drivers of acute hunger for 4.4 million Haitians [54].

Extreme events affect human health in various ways, including via the spread of vector-borne and waterborne infectious diseases, as well as damage to food systems [5]. A meta-analysis of recent drought studies put in evidence that drought conditions increase the odds of wasting and underweight by impacting crop production and food availability [57]. Analyses of data from the International Disasters Database and the Global Expanded Nutrient Supply model for the period of 1961–2010 showed that the effect of an extreme weather event was mostly magnified among landlocked developing countries and low-income food-deficient countries, with significant nutrient supply changes ranging from −1·61 to −7·57% of the average supply [58]. Overall, rising temperatures and increases in the frequency of extreme events threaten global food security, with a 1.8–5.6% reduction in global yield potential for major crops observed from 1981 to 2019. In particular, crop yield potential has followed a consistently downward trend for maize (5.6%), soybean (4.8%), winter wheat (2.1%), and rice (1.8%). Food supply to the population is a strategic priority of governments. In LMICs, this represents an acute issue due to the inability to saturate the inner food market through domestic agricultural production. Thus, the global food supply is essential to avoid hunger, malnutrition-related diseases, and social instability [59].

Besides extreme weather events, the global agricultural market is also threatened by the Russian–Ukrainian conflict currently underway. Ukraine supplies more than 14% of the global food market, including wheat (12.5%), corn (12.8), sunflower oil (47%), and sunflower meal (54%). The war is destroying the Ukrainian agrarian sector and food stocks (in 2022, grain yield is expected to decrease by 10–15%), with catastrophic implications for the national economy and food security throughout the world, leading to a drop in agri-food deliveries to Asia and Africa amounting to 49% and 13%, respectively.

### 3.3. The COVID-19 Pandemic

The frequency of zoonosis emergence such as the COVID-19 pandemic can originate from environmental changes as a result of rapid unplanned urbanization, agricultural expansion, intensifying livestock production, increases in population density, and loss of biodiversity [3,60]. More than four years of progress against poverty have been erased by the COVID-19 pandemic [61], demonstrating the fragility of current food systems [29]. Worldwide and particularly in fragile contexts, malnutrition and food insecurity have been worsened by the COVID-19 pandemic due to an increase in unemployment and a decline in household incomes; constraints on the availability and affordability of nutritious foods; interruptions of health, nutrition, and protection services; and limited opportunities for physical activities [61,62,63,64,65,66]. School closures, movement restrictions, and nationwide lockdowns impacted food systems by disrupting the production, transportation, and sale of fresh, nutrient-rich, and affordable foods, which, in turn, led to price volatility and forced millions of families to rely on low-cost, nutrient-poor alternatives, thus influencing children’s dietary intake through changes in their home food environment [67]. Notably, in LMICs, the price of some essential foods increased significantly, affecting their accessibility [68,69]. Vulnerable populations, such as children and women of low SES, are the most affected [62]. In drought-affected area, the COVID-19 pandemic, climate change, and the recent wheat crisis created a “perfect storm” of malnutrition in children and breastfeeding mothers [70]. The World Food Programmes Annual Review 2021 estimated an extra 47 million women pressed into extreme poverty by the pandemic from 2019 to 2020 [71]. According to the latest UNICEF report, the number of children suffering from malnutrition is rapidly rising, where the risk of child mortality is already the highest. In the meantime, the COVID-19 pandemic and armed conflict are driving up the prices of ready-to-use therapeutic food, getting harder to respond effectively [72]. The impact of the COVID-19 pandemic on early-life nutrition is expected to have long-term consequences for childhood growth, development, and chronic disease risk [73]. Based on moderate estimates of the potential impacts of the pandemic-triggered crisis on undernutrition among children under 5 years, nearly 2.6 million additional children will be stunted in 2022, and the number of children suffering from wasting will increase by an added 9.3 million, with approximately 168,000 additional deaths [74]. As an indirect effect of the COVID-19 pandemic, increased childhood obesity is also assumed. Exposure to COVID-19-related measures, leading to increased food insecurity and decreased physical activities, along with reduced access to nutrition education, is hypothesized to strongly impact childhood obesity risk factors and psychosocial stressors [64]. COVID-19-related stress was found to be associated with increased non-nutritive consumption of sweet and savory food and snacks [75].

## 4. Food Systems and Sustainable Nutrition Early in Life

In the last decade, a thriving body of literature on food systems proposed different types of frameworks to explain issues related to food and nutrition security [7,16,60,76,77,78,79]. Common aspects of these food system approaches are a focus on drivers and determinants that shape them from a social, economic, and environmental perspective (Box 1).

Box 1 **Conceptual frameworks of food systems and political economy.**

When considering drivers, conceptual frameworks of food systems draw the attention to climate change, globalization and trade, income growth and distribution, migration and population growth, and social and cultural norms. On the determinants side, scholars and policymakers have highlighted specific factors that affect eating patterns, such as food supply chains, the food environment (retailers, commercial markets, informal vendors, etc.), the household environment, and the behavior of caregivers.

To date, only UNICEF and GAIN’s Innocenti framework have linked food systems to children’s and adolescents’ well-being, putting their diets at the heart of analyses of the drivers and determinants [16].

Works carried out by the Lancet Commission represent valuable milestones in food system analysis. In 2019, EAT-Lancet set out the parameters of a“sustainable diet” in anattempt to inform decisions on policies that would influence the determinants and drivers of food systemsrelated to health and the environment [80].

The Lancet’s *The Global Syndemic of Obesity, Undernutrition, and Climate Change,* went further by adopting a political economy lens to rethink the systems of food and agriculture, transportation, urban design, and land. The Commission cast light on the “policy inertia” that characterizes the current governance structure skewed towards the disproportionate power of multinational food and beverage corporations at the expense of health- and environment-supportive policies [7].

The trade–food system–nutrition–climate nexus highlights the perverse effects of trade instruments related to agriculture and the food industry and their impacts on diets. Well-known examples are the spread of ultra-processed food through foreign direct investment by multinational companies into local production between the 1980s and 2000, representing a period of extensive investment and trade liberalisation around the world [81,82]. This strand of literature argues that private trade and investment tools accrue benefits for food availability and diet quality, complementing areas of domestic policies. These can include investments in domestic value chains for nutritious products and social safety nets. Investments in local production are especialy important for import-dependent LMICs because they are more prone to food insecurity and malnutritionand are vulnerable to the volatility of global commodity prices [83].

International demand for agriculture commodities represents the second leading global source of greenhouse gas emissions and drives tropical deforestation and biodiversity loss. Between 2010 and 2014, trade in agriculture commodities accounted for 29–39% of deforestation-related emissions, with livestock and oilseed production representing over half of this amount [83].

The relations between food systems, nutrition, and the climate are complex, with environmental changes acting as both a driver and an outcome of food systems [29]. Food and agriculture, transportation, urban design, and land use are the major systems driving the global syndemic [7,80]. The current unsustainable patterns of food consumption and production engender the triple planetary crises of climate change, biodiversity loss, and pollution. Agriculture occupies approximately 40% of global land, and food production is responsible for up to 30% of global GHG emissions and 70% of freshwater use [80]. From 2000 to 2016, GHG emissions increased by 14% from livestock and by 58% from poultry [4]. Existing agriculture systems rely on an overabundance of staple grains (i.e., rice, wheat, and maize) rather than on a broader range of more diverse and healthier foods such as fruits, nuts, and vegetables. Despite consuming 83% of agricultural land and being responsible for 60% of agricultural GHG emissions, livestock farming provides only 18% of total food energy [84]. Highly processed foods are available, cheap, and intensively marketed both in HICs and in LMICs. Due to the increasing pressure from agriculture and industry, demand for water is rising [61]. In addition, an excessive amount of food continues to be lost (13.3%) after harvesting and before reaching retail markets or is wasted (17%) at the consumer level, translating to 121 kg per person each year [61]. With food production causing major global environmental risks, sustainable food production, i.e., a fundamental shift in production priorities, is needed to safeguard biodiversity and substantially reduce substantially water and land use, as well as greenhouse gas emissions [80].

As underlined by Hollis and colleagues [27], there is an urgency to transform food systems, starting with children’s first foods. Failing to improve breastfeeding rates, i.e., to reduce formula feeding, may harm economies and environments [85]. In contrast to the environmental costs and negative impacts of infant formula, breast milk is definitely an optimal sustainable food source with zero environmental footprint or food waste. According to estimates that the production of 1 kg of milk powder uses 4700 L of water and emits 21.8 kg CO_2_-eq of GHG, exclusive breastfeeding could save 105,280 L of water and 488 kg CO_2_-eq. Furthermore, the environmental cost of infant formula includes the deforestation of land and loss of biodiversity, the extensive use of materials for packaging, the high-demand use of energy resources in manufacturing, and GHG emissions from transportation. With respect to complementary and young child feeding, reducing the overall production and overconsumption of ultra-processed foods (contributing 30–50% of total energy intakes in some HICs, with sales increasing in middle-income countries), while increasing consumption of minimally processed foods may concomitantly improve diet quality and reduce environmental footprints by diminishing the amount of resources used in manufacturing, packaging, and distribution. Attaining healthy diets would lower food-based GHG emissions by 30% compared to current intakes [27].

**Figure 2:**
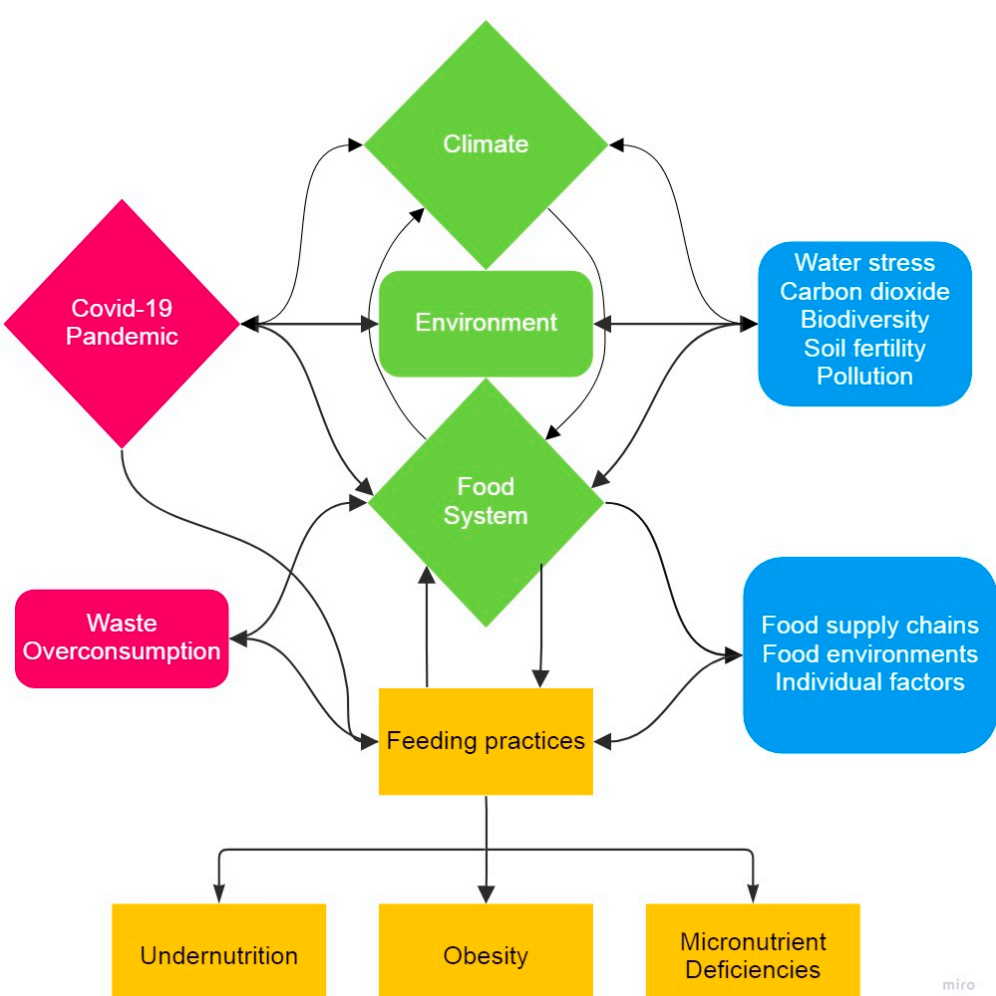
The interrelated inputs, outcomes, and feedback between the food system, climate, and the COVID-19 pandemic. Through their impact on environmental outcomes (i.e., biodiversity and soil, air, and water quality), climate inputs (i.e., weather patterns and temperature) influence food systems in terms of food production and security and, ultimately, feeding practices. Conversely, food systems (i.e., patterns of food production and consumption) affect both human and environmental health outcomes. The COVID-19 pandemic has amplified the weaknesses of the food system–climate–health net.

The interconnectedness between food systems and the climate, including the existence of feedback, is summarized in Figure 2.

### 4.1. IYCF Practices and Food Systems: Existing Gaps

Children aged 0–24 months have very specific dietary needs to foster quick growth and development. Therefore, policies related to food systems must be analyzed and designed considering that this age group represents a small percentage of a stable population and, accordingly, their diets have only a minor impact on food systems and food production. We argue that some elements of existing food system frameworks may not be fully applicable when designing policies aimed at improving the diets of children aged 0–2 years. Therefore, we propose the following questions for future research: *To what extent is the concept of a sustainable diet applicable to all age groups? To what extent are market mechanisms able to influence the demand and supply of recommended complementary feeding?*

#### 4.1.1. Sustainable Diets and IYCF

The concept of a sustainable diet came to prominence during the 1980s and is based on the idea that healthy consumption patterns work as a driver of sustainable production and may lower environmental pressure. Although parents can play a key role in influencing children’s food choices towards sustainable diets from the very early stages of life [86,87], the dietary requirements for children aged 6–24 months are unique. Animal-source foods, including red meats, play a key role in child growth improvement, micronutrient status, cognitive performance, and motor development. In promoting a reference sustainable diet, the EAT-Lancet commission draws attention to specific needs according to both age group and geographical area, as in the case of sub-Saharan Africa, where low consumption of animal-source protein may determine multiple micronutrient deficiencies, anemia, and stunting [80]. *To what extent and in which areas of the globe do nutrition quality and low environmental impact have a greater chance to exert combine effects according to the concept of a sustainable diet?* Ninety countries have developed food-based dietary guidelines; however, these are often not specific to the different phases of children’s growth and do not consider the characteristics of food systems according to the geographical area [16]. Analytical research has been conducted to explore quantitative methods to define the indicators of sustainable diets [88,89,90]. However, there exists a range of different methodologies, making them sometimes difficult to apply [91].

#### 4.1.2. Market Mechanisms and IYCF

The Lancet Commission report on the Global Syndemic explained how obesity, undernutrition, and climate change inter-relate with each other, as they are immersed in complex systems in which different actors and dynamics coexist [7]. This complex web of interactions is at the heart of the system of five main domains, also called feedback loops (business, demand and supply, health, ecology, and governance), which are able to create both virtuous and vicious outcomes for health and the environment. However, the feedback loops related to business and supply and demand may not be fully applicable to children aged 0–24 months because the weak demand to fulfill complementary feeding recommendations does not create an incentive for the food industry (whether a multinational or small–medium enterprise) to produce and deliver the recommended food. Similarly, children in this age group are not able to influence the demand and the supply of complementary feeding because they do not participate directly in the market. This is notably true in poverty-stricken areas described in the literature by the terms “food swamp” or “food desert” [92,93]. These terms indicate marginalized areas in HICs and LMICs populated by fast-food chains, food outlets, informal food vendors, and traditional markets selling cheap ultra-processed foods and the food basket for a household is more likely to be composed of essential food items scarce in animal-source protein due to families’ economic constraints [30]. In these settings, caregivers’ scarce knowledge of nutrition is also less likely to create an incentive for children to consume recommended foods.

Specific policies aimed at improving child feeding practices should carefully consider the extent to which they should rely on existing market mechanisms.

## 5. Approaches and Strategies

Child malnutrition has not only to be treated but also prevented. It is crucial to build the resilience of communities hit hardest to reverse the global hunger spike [94]. Governments and donors must maintain child nutrition as a priority and support resilient systems [73]. Early-life nutrition, dietary diversity, sustainable food environments, empowerment of women, urban design and land use, and socioeconomic factors are considered the foundation on which to redesign an overarching strategy. These actions are delivered through platforms both within and outside of health facilities and cross-cutting sectors to nutrition, notably education, agriculture, food systems, social safety nets, and WASH (water, sanitation, and hygiene) [95,96].

Existing actions focused on undernutrition likely have the potential to be scaled-up for double-duty or triple-duty actions with the aim of simultaneously tackling the multiple components of the global syndemic, i.e., promoting healthy growth in early-life and sustainable culturally appropriate diets throughout the life course [97]. Notably, effective strategies are needed during pregnancy, in addition to support for optimal IYCF while counteracting the marketing of breast milk substitutes [27]. These include increasing funding for breastfeeding; enacting better family leave and workplace policies; improving the quality of maternity facilities and access to skilled lactation counseling; strengthening community networks; and creating a monitoring system to track the progress of policies, multilateral programmes, and funding [25]. During complementary feeding, encouraging consumption of diverse minimally processed plant foods with appropriate amounts of animal-sourced foods, i.e., a dietary shift towards a more sustainable and healthy dietary pattern, such as the Mediterranean diet, may play an important role in optimizing carbon footprints and preventing obesity [80,98]. Climate adaptation measures in agriculture, livestock production, and fisheries need to be increasingly adopted in both LMICs and HICs in order to promote food and nutrition security, as well as livelihoods. Strategies drawn from conservation agriculture, agroforestry, and agro-ecology may have the potential to mitigate GHG accumulation in the atmosphere, reverse degradation caused by conventional farming (reduced soil disturbance and fertilizer use), increase crop diversity, and promote water-saving techniques. This approach boasts research on crop varieties, animal breeds or fish, and forest species that are required both in response to changing climate conditions and for the promotion of biodiversity [41,99]. Governments need to focus on supporting the poorest farmers to transfer knowledge with respect to climate adaptation strategies, as well as trends in temperature and precipitation. This can be achieved by setting up appropriate platforms and technologies for information sharing through early warning systems (e.g., FEWS NETS) so that farmers can make informed decision in light of the occurrence of extreme weather events [100].

A recalibration of food systems is mandatory for the sake of global warming, planetary sustainability, and human health [80]. The COVID-19 pandemic has highlighted the fragility of the current system. Reinforcing local and regional supply chains helps reduce the risk of food reserve shortages that rely on the global market [101,102]. Moreover, policy makers must strengthen compliance with food safety standards throughout food chains and provide specific support to small farmers and small–medium enterprises [103,104]. Whereas the market fails to address food and nutrition insecurity, social safety nets are needed to protect vulnerable swaths of the population based either on food aid or cash-based transfer that not only privilege calorie intake but also healthy food [105]. Finally, pricing policies (e.g., subsidizing unprocessed or minimally processed plant foods while taxing ultra-processed foods) and regulatory measures aimed at protecting children from aggressive food marketing may be effective approaches to address food systems and drive people’s choices [27].

## 6. Conclusions

The world is not on track to end malnutrition and poverty by 2030. A confluence of crises is impacting the world, with the pandemics of obesity, undernutrition, and climate change representing severe threats to child health. Nutrition, health, and the natural environment are closely linked across the life course. Frequent disasters resulting from climate change and pandemics weaken food systems and exacerbate food insecurity worldwide, and current agri-food systems significantly impact the environment, the climate, and, ultimately, child feeding practices and health. Persistent inequalities in childhood nutrition exist, suggesting that a more holistic approach is paramount to guarantee equity and healthy environments, i.e., to achieve sustainable permanent solutions in the real world. Early-life nutrition, food environments, and socioeconomic factors must be considered as the basis to further scaleup ongoing initiatives. Hence, systematic interventions and policies that create healthy, sustainable, and diverse food systems are needed. A massive transformation of the food system is mandatory to tackle climate change and to save and improve children’s lives and futures.

## Data Availability

Not applicable.
